# Underwater Image Enhancement via Multiple-Enhanced-Layers Fusion and Transmission-Driven Color Restoration

**DOI:** 10.3390/s26175663

**Published:** 2026-09-06

**Authors:** Zhengmao Li, Chi Zhang, Yanping Chen, Jun Zhang

**Affiliations:** 1 School of Artificial Intelligence and Big Data, Hefei University, Hefei 230601, China; lizm@hfuu.edu.cn (Z.L.); hfuu_zc@hfuu.edu.cn (C.Z.); 2 School of Artificial Intelligence, Anhui University, Hefei 230601, China; junzhang@ahu.edu.cn

**Keywords:** image restoration, pixel-based transmission estimation, transmission-driven color restoration, underwater image enhancement

## Abstract

Underwater images captured by sensors suffer from low contrast and blurry details due to the interference of light absorption and scattering in underwater scenes. Good visibility restoration is often desired for practical processing applications. Current image enhancement methods often rely on prior assumptions to reconstruct a clear image without considering the inherent correlation of underwater image degradation, introducing unconsiderable enhancement results. Thus, this paper proposes an underwater enhancement method based on multiple enhanced layers fusion and transmission-driven color restoration, named EFCR, which consists of three key modules: a pixel-based transmission computation (PTC), a multiple-enhanced-layers fusion (MELF), and a transmission-driven color restoration (TCR). First, PTC designs a linear transformation to adjust the saturation and estimates the transmission based on the mapping relationship between the transmission, the brightness, and the saturation, preventing the transmission from being under-estimated. Then, MELF extracts the original details from the luminance channel and enhances these desired details based on the estimated transmission. Meanwhile, adaptive histogram equalization is used to improve the global brightness. Finally, TCR further analyzes the inherent correlation between the transmission and the image degradation, and constructs a compensation factor to adaptively correct the attenuated *a* and *b* channels of Lab space, producing a good enhancement result with reasonable brightness and natural colors. Extensive experiments on three underwater image datasets demonstrate the effectiveness and robustness of the proposed method in underwater image restoration. Especially, the average $\bar{r}$ and Blur values of our method at most incline and decline by 99.87% and 10.22%, respectively, which shows our method has obvious advantages in edge enhancement and haze removal. Moreover, our method provides helpful support for color restoration and image salient detection, and also shows good generalization capability for enhancing outdoor hazy images.

## 1. Introduction

Image sensors play an increasingly important role in industry, agriculture and daily life. However, sensor images often show poor visual effects in underwater conditions, i.e., ow contrast, blurry details and unnatural colors. High quality images are of great significance to many real-time applications for underwater scenes, such as vehicle detection, visual tracking, etc. However, the light is strongly scattered and absorbed in scattering scenes, and the visibility quality of underwater images is typically characterized by low contrast and blurry details. These degradation issues provide poor support for the application performance of them in underwater scenes.

Currently, a large number of image enhancement and restoration methods [1,2,3,4,5,6,7,8] are introduced to improve the visibility quality of sensor images, and these methods can be roughly divided into three classes: imaging model-based restoration methods, model-free enhancement methods, and deep learning-based methods. Imaging model-based restoration methods build the physical imaging model by simulating the light propagation process, and find diverse priors to calculate the key parameters, i.e., the transmission and the airlight, etc., improving the image quality. For instance, He et al. [9] proposed a dark channel prior, which reveals the r, g and b channels have extremely low values tending to zero for a local non-sky patch, thereby estimating the transmission based on patch-based operations. Inspired by DCP, Meng et al. [10] further refined the coarse transmission of DCP using a regularization strategy, and produced good enhancement results. In summary, these methods usually consume a lot of time to compute the dark channel, causing low efficiency. Meanwhile, the results easily generate halo artifacts due to the patch-based operations. Model-free enhancement methods enhance the visibility quality of the hazy images by revising the pixel values, which consist of histogram equalization, image fusion, and variational-based methods. For instance, Ancuti et al. [1] first yielded two enhanced versions of hazy images using white balance and contrast stretching methods, and then fused the two images based on a multi-scale fusion strategy to generate a good result. Yu et al. [4] proposed ZAP, which symmetrizes the histograms of the *a* and *b* channels in the CIELab space and adjusts their dynamic ranges to correct color distortion. Mi et al. [7] introduced a generalized enhancement framework for hazy images with complex illumination. Zhang et al. [8] combined attenuation-map-guided color correction, global and local contrast enhancement, and weighted wavelet visual-perception fusion. Zhuang et al. [11] improved the image quality by integrating a hyper-Laplacian reflection prior into a retinex-inspired variational model, and this method significantly enhanced the contrast of complex images while effectively reducing the noise, but the computation complexity is high. Usually, it is difficult to solve the model parameters due to the sophistication and variety of the objective function, and the subjective and objective results are often inconsistent. Deep learning-based methods use the strong computation efficiency of GPU hardware to learn the mapping relationship between the hazy image and the clear image. For example, Liu et al. [2] proposed Rep-UWnet, a lightweight network constructed with a fully convolutional architecture and densely connected RepConv blocks. Zhao et al. [5] developed an MDCNN-VGG model that uses multiple DCNN branches and a VGG network to improve multi-domain adaptation. Li et al. [6] introduced LCS-Net, which combines learnable color correction with selective multi-scale feature fusion. Cai et al. [12] proposed a dehazing network, named DehazeNet, to extract features for estimating the medium transmission. However, the experimental results show some residual haze. Li et al. [3] achieved image enhancement via an all-in-one framework, called AOD-Net, to calculate the parameters within a reformulated atmospheric scattering model, thereby optimizing the parameter estimation. These deep learning frameworks produce good performance on synthetic datasets, but the robustness and effectiveness of their enhancement results are highly correlated to the training data. Since there is a significant gap between the synthetic and real-world images, their generalization performance does not work well in real underwater scenes.

To deal with these challenging problems, we propose an image restoration method, called EFCR, which contains a pixel-based transmission estimation (PTE), a multiple-enhanced-layers fusion (MELF), and a transmission-driven color restoration (TCR). PTE provides a fast transmission estimation using the relationship between the transmission, the brightness, and the saturation. MELF amplifies the details in the gradient domain and expands the global brightness using adaptive histogram equalization. TCR suggests a color restoration based on the estimated transmission. Our method fully considers the advantages of both physical-model-based and model-free categories, and the inherent correlation of underwater image degradation, which can adapt to various water types. We summarize the contributions of this paper as follows.

- We propose an effective and robust underwater image enhancement method. The extensive experiments on three underwater image datasets reveal stable enhancement performance under various scenes. Meanwhile, it shows fine salient detection results on the enhancement results and good generalization performance on other degraded images.
- We present an effective transmission estimation method based on pixel-based operation, which first designs an adjustment coefficient based on the correlation between the saturation and the brightness, slightly revising the saturation, and then estimates the transmission using the relationship between the transmission, the brightness, and the saturation.
- We introduce a global details and brightness enhancement framework via the transmission and adaptive histogram equalization, which uses the reciprocal of the transmission to enhance the details in the gradient domain and applies adaptive histogram equalization to address the low illumination of underwater images.
- We propose a color restoration method based on the estimated transmission, which analyzes the high correlation between the transmission and color degradation. Based on this consideration, we build an adaptive compensation factor to dynamically compensate for the *a* and *b* channels that affect the colors, achieving color restoration.

## 2. Related Work

Over the past ten years, related researchers have proposed a large number of image enhancement and restoration works to improve the visibility of underwater images, and these works can be generally divided into three groups: physical model-based methods, model-free methods, and deep learning-based methods. This section will briefly introduce the related works.

### 2.1. Physical Model-Based Methods

Physical model-based methods strictly rely on the construction of underwater optical imaging models. Then, these methods usually use hand-crafted priors and assumptions to calculate key parameters, i.e., underwater scene transmission and backscatter light, thereby reversing the physical model to generate clear images. For example, He et al. [9] proposed a representative Dark Channel Prior based on the statistical knowledge of many outdoor clear images, and this method has been proven to be useful for outdoor hazy scenes. However, it is less effective for underwater images. To enhance its adaptation, Drews et al. [13] only use blue and green channels to calculate the transmission while considering the attenuation characteristics of the red channel. The experiments showed that this revision can enhance underwater image quality to some extent. Similarly, Galdran et al. [14] compensated for the high attenuation of red light based on an automatic red-channel image restoration method. In recent years, Ning et al. [15] designed an adaptive parameter optimization strategy and an adaptive color constancy method to estimate the backscatter light. Meanwhile, to eliminate the effects of halos and blurry edges, they also applied a smoothness and uniformity transmission computation method, achieving state-of-the-art performance. Stephan et al. [16] proposed a problem-specific physical model of underwater environments and introduced a comprehensive method to produce the inversion of degradation processes, obtaining high-quality images. However, these methods suffer from notable limitations: the parameter estimations heavily depend on prior assumptions, and the dynamic and complex underwater scenes often violate the statistical rules of these knowledges, causing suboptimal enhancement performance.

### 2.2. Model-Free Methods

Model-free methods often use histogram equalization, image fusion, and retinex-inspired strategies to improve visual quality, rather than relying on the physical imaging model. For example, Iqbal et al. [17] presented an integrated color model method, and this method first used contrast stretching to achieve the balanced histogram regarding the red and green channels of RGB color space, then applied a similar operation to control the saturation and intensity components of HSI space. To deal with the noise amplification of standard histogram equalization, Garg et al. [18] used a contrast-limited adaptive histogram equalization to improve the overall quality. Ancuti et al. [19] proposed an image fusion method to fuse a color-corrected image and a contrast enhancement image according to the given weight maps, producing the desired results with high contrast and good colors. More recently, Liu et al. [20] used histogram similarity cues to achieve adaptive information transfer, and linear stretching was used to adjust the dynamic range. Then, a superior result was output based on a perception fusion strategy. Although model-free methods have advantages such as simple implementation and low computation complexity, their enhancement results also show distorted colors and over-enhanced contrast due to ignoring the inherent correlation between underwater image degradation degree and scene depth. Thus, to further improve the enhancement performance, researchers should consider combining the advantages of physical model-based and model-free methods.

### 2.3. Deep Learning-Based Methods

Deep learning-based methods directly learn nonlinear mappings from the paired degraded images and the clear images based on neural networks. For example, Li et al. [21] utilized synthetic underwater images to train an end-to-end CNN framework, achieving notable improvements in correcting the colors and enhancing the contrast. However, the performance of this method heavily relies on the quality of synthetic data. Zhu et al. [22] introduced a U-shaped hybrid network, which mainly includes a color correction module and a fused feature-guided cross-attention block. This network can simultaneously obtain local textures and global scene structure. Liu et al. [23] constructed an image enhancement method by fusing a CNN-attention bottleneck layer and a Transformer-based channel attention model and designed a new loss function to correct the contrast and chromatic aberrations. Li et al. [24] applied a physical method to simulate underwater scenes, generating ten types of underwater images. Meanwhile, they proposed the UWCNN model to enhance underwater scenes based on these synthesized underwater images. However, this method showed some limitations in enhancing complex underwater scenes. Except for CNNs, GAN-based methods also achieve good performance in underwater image enhancement. For example, Zhou et al. [25] proposed an underwater generative adversarial network based on multiple color spaces, named MCS-UGAN, which consists of two main modules: a deblurring module and a color correction module. The former applied multi-scale attention on the Y channel of YUV space, and the latter used a U-Net containing residual blocks in RGB space. Lei et al. [26] presented UIGAN to improve underwater image quality, which produces large-scale paired images by integrating the attenuation coefficients into a simplified physical model, and their proposed STLNet performs well in enhancement. Ding et al. [27] used polarization imaging to capture multi-polarimetric images, and proposed multi-polarization fusion generative adversarial network to rebuild the clear scenes, which take into account the polarization states of underwater physical imaging. Liu et al. [28] constructed a conditional GAN to correct the color and contrast based on multilevel feature fusion, which improves the color. Most deep learning methods need a mass of paired data, but it is extremely difficult to generate high-quality paired datasets in complex and dynamic underwater environments.

## 3. Proposed Methodology

**Figure 1:**
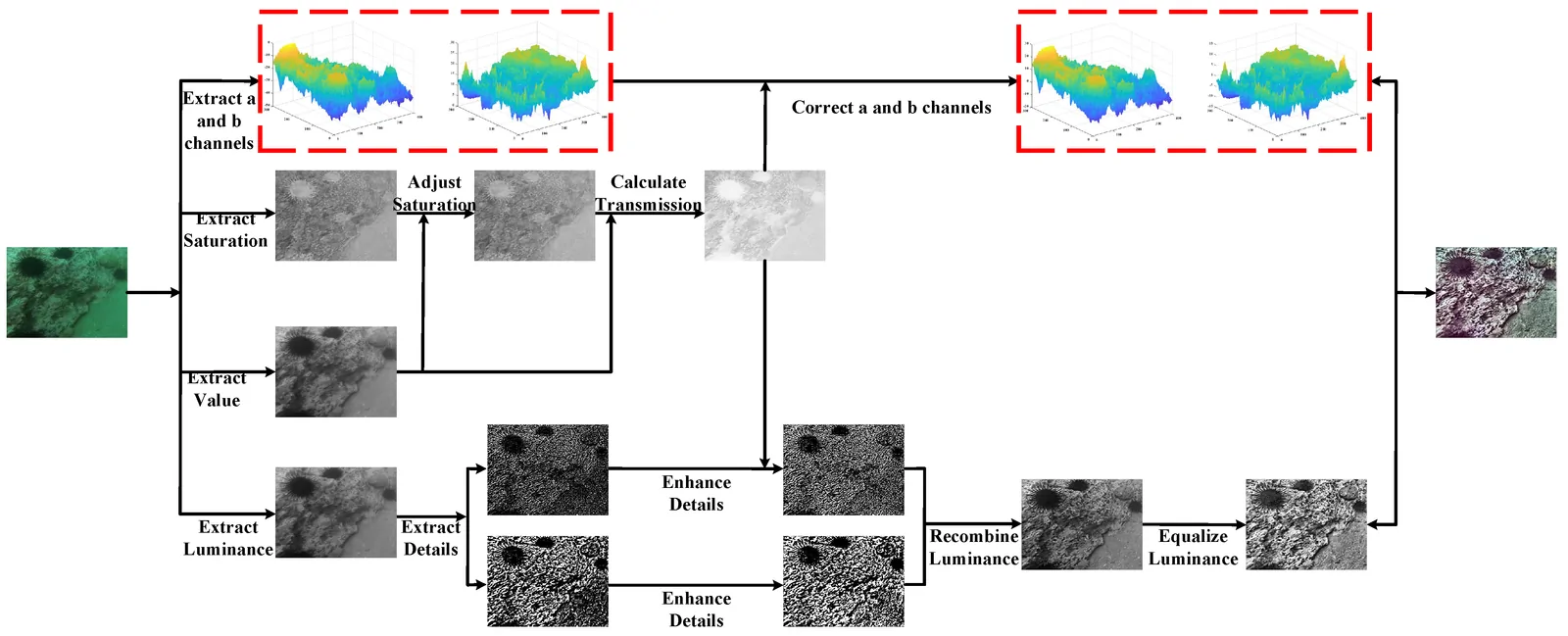
Flowchart of our EFCR. We first use the brightness and the saturation to compute the adjustment parameter, and slightly revise the saturation and produce the transmission. Then, we apply the estimated transmission to amplify the details. Meanwhile, adaptive histogram equalization is used to enhance the global brightness. Finally, we correct the color casts relying on a function only of the transmission, thereby producing good enhancement results.

The overall framework of our method is illustrated in Figure 1. The middle, lower and upper branches of the figure correspond to pixel-based transmission estimation (PTE), multiple-enhanced-layers fusion (MELF), and transmission-driven color restoration (TCR), respectively. PTE estimates the transmission from the HSV saturation and value channels. MELF uses this transmission to enhance the detail layers extracted from the Lab luminance channel, followed by luminance recombination and adaptive histogram equalization. TCR uses the estimated transmission to correct the Lab $a^{*}$ and $b^{*}$ chrominance channels. Finally, these corrected channels are fused with the enhanced luminance channel to generate the output image.

### 3.1. Pixel-Based Transmission Estimation

Most remote sensing image restoration methods often use the atmospheric scattering model to rebuild the clear image, which can be expressed as:

(1) $$I_{c}\left(x,y\right)=J_{c}\left(x,y\right)t_{c}\left(x,y\right)+A_{\infty }\left(1-t_{c}\left(x,y\right)\right).$$

where *c* is the color channel and c∈{r,g,b}. I(x,y) and J(x,y) are the input hazy image and the clear image, respectively. t(x,y) and $A_{\infty }$ are the medium transmission and the global airlight, respectively. (x,y) is the pixel coordinate of the input image. As can be seen from Equation (1), to obtain the clear image *J*, we need to estimate the transmission *t* and the global airlight $A_{\infty }$. Among the current methods, DCP-based methods are widely used to solve the two key parameters. However, these methods have two limitations: (1) the dark channel is computed by patch-based operation, which is often time-consuming; (2) the restoration results contain a lot of halo artifacts. To overcome these issues, we propose a pixel-based transmission estimation method to accelerate the running time and reduce the halo artifacts.

**Figure 2:**
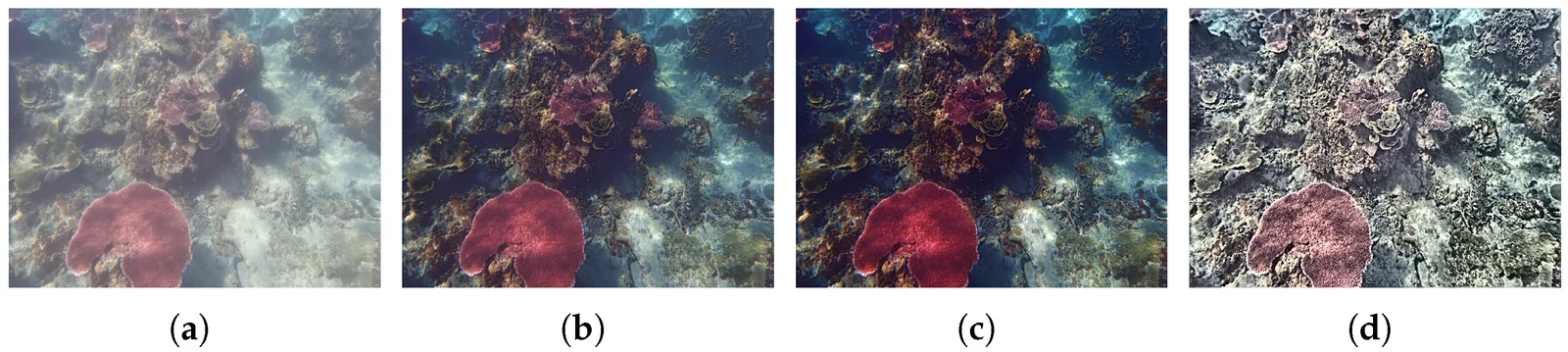
Comparative results of different methods. From (**a**–**d**): the raw image, the restoration result of Lu et al. [29], Lu et al. with our transmission, and our full method. As can be observed, combining the method of Lu et al. with our transmission can obtain a more clear image, and our full method can produce a good result with a natural appearance and high contrast.

Lu et al. [29] deduced the relationship between the transmission, the saturation, and the brightness based on the dark channel prior, and this relationship can be mathematically expressed as follows:

(2) $$\tilde{t}\left(x,y\right)=1-\left(1-\tilde{S}\left(x,y\right)\right)V\left(x,y\right),$$

where V(x,y) denotes the brightness channel of the input image and $\tilde{S}\left(x,y\right)$ is a revised saturation for avoiding under-estimation of the transmission, and it can be formulated as follows:

(3) $$\tilde{S}\left(x,y\right)=\min\left(S^{a}\left(x,y\right)+\beta ,1\right),$$

where S(x,y) denotes the original saturation, 0<α<1, 0<β<1. Figure 2b shows the restoration result of Lu et al., and we can see that the result contains some residual haze due to the insufficient adjustment of the saturation. Thus, to further improve the visibility quality, we propose a more accurate saturation revision strategy. Firstly, in the HSV color space, the saturation S(x,y) and the brightness V(x,y) are defined as:

(4) $$S\left(x,y\right)=1-\frac{\min_{c\in \{r,g,b\}}I_{c}\left(x,y\right)}{\max_{c\in \{r,g,b\}}I_{c}\left(x,y\right)}=1-\frac{I_{c,\min}\left(x,y\right)}{I_{c,\max}\left(x,y\right)},$$

(5) $$V\left(x,y\right)=\max_{c\in \{r,g,b\}}I_{c}\left(x,y\right)=I_{c,\max}\left(x,y\right).$$

Based on the observation of Equations (4) and (5), we can see that the compensation of the saturation is highly correlated with the brightness. Inspired by this, the average correlation coefficient between the S and V channels can then be computed as follows:

(6) $$\rho =\frac{\sum_{(x,y)\in I}\left(M_{V}\left(x,y\right)\times M_{S}\left(x,y\right)\right)}{HW\times \sqrt{\mathrm{std}\left(V\right)\times \mathrm{std}\left(S\right)}},$$

where std is the standard deviation and *H* and *W* denote the height and width of the input image, respectively. $M_{V}$ is the square of the difference between the V channel and the mean value of the V channel. $M_{S}$ is the square of the difference between the saturation channel and the mean value of the saturation channel. $M_{V}$ and $M_{S}$ can be expressed as follows:

(7) $$M_{V}\left(x,y\right)={\left(V\left(x,y\right)-\bar{V}\right)}^{2},$$

(8) $$M_{s}\left(x,y\right)={\left(S\left(x,y\right)-\bar{S}\right)}^{2}.$$

With the help of Equation (6), we use the following equation to optimize the saturation based on refs. [29,30]:

(9) $$\tilde{S}\left(x,y\right)=\min\left(S\left(x,y\right)+\alpha \left(V(x,y)-\rho \times V(x,y)\right),1\right).$$

Finally, we can easily estimate the transmission using Equation (2). To demonstrate the effectiveness of our method, the transmission estimation of Lu et al. is replaced by our method, and Figure 2c shows the corresponding restoration result. As shown, the haze can be completely removed and the contrast is sufficiently enhanced.

### 3.2. Gradient Domain-Guided Contrast and Detail Enhancement

Most IFM-based methods often build hand-crafted priors and assumptions to calculate the waterbody parameters, and the restoration results inherently depend on the effectiveness and robustness of the parameters. However, these conditions are frequently violated in variable and complex underwater scenes, thereby causing unwanted noises and color casts. Thus, we attempt to propose an underwater image restoration framework by joining the advantages of model-based and model-free methods, reducing the strict reliance on the accuracy of parameter estimations. The relationship of the contrast between the degraded and the clear images is expressed as:

(10) $$\sum_{\left(x,y\right)}∥\nabla I\left(x,y\right)∥=t\sum_{\left(x,y\right)}∥\nabla J\left(x,y\right)∥\leq \sum_{\left(x,y\right)}∥\nabla J\left(x,y\right)∥,$$

where ∇ denotes a gradient operator and ∇I and ∇J indicate the gradients of the input image and the clear image, respectively. Equation (10) implies that the degradation of the gradient is highly correlated with the transmission, and the gradient of the clear image can be increased by amplifying the gradient of the degraded image based on the reciprocal of the transmission. According to this foundation, we use a contrast enhancement strategy to simultaneously improve the contrast and the details in the multi-scale gradient domain. Concretely, we first divide the luminance channel $I_{L}$ of the input image into a base layer and two residual detail layers using a weighted least squares (WLSs) filter, and this WLS filter can be written as follows:

(11) $$u=F_{\phi }\left(L\right)={\left(U+\phi G_{L}\right)}^{-1}L,$$

where *u* denotes the filtered image of $I_{V}$ and *U* indicates the identity matrix. $G_{L}=D_{i}^{T}A_{i}D_{i}+D_{j}^{T}A_{j}D_{j}$, where $D_{i}$ and $D_{j}$ represent the discrete differentiation operators; $A_{i}$ and $A_{j}$ are the matrices containing the smoothness weights. By operating the ϕ value, we can produce a set of smoothed images $u^{1},u^{2},\ldots ,u^{k-1}$, and the smoothest image $u^{k-1}$ is selected as the base layer $b_{L}$. Specifically, the discrete differentiation operators $D_{i}$ and $D_{j}$ compute first-order differences between vertically and horizontally adjacent pixels, respectively. Operating the ϕ value means varying the WLS regularization parameter: a larger ϕ imposes stronger smoothing. In the implementation, $\phi_{1}=0.1$ and $\phi_{2}=1.0$ are used to obtain a mildly smoothed representation $u^{1}$ and a more strongly smoothed representation $u^{2}$, respectively. The two residual detail layers are yielded as:

(12) $$d_{i}=u^{i-1}-u^{i},\;\mathrm{where}\;i=1,\ldots ,k-1\;\mathrm{and}\;u^{0}=I_{V},$$

where k=3 in our paper. It comprises the original luminance representation $u^{0}$ and the two WLS-smoothed representations $u^{1}$ and $u^{2}$, producing two residual layers $d_{1}$ and $d_{2}$. This fixed setting provides one fine-scale and one coarser-scale detail layer with a compact decomposition. According to Equation (10), as the detail layers $d_{i}$ are obtained, we can generate the enhanced gradient of the clear image based on the estimated transmission *t*, which can be computed as follows:

(13) $$\nabla d_{i}^{\prime }=\frac{\omega_{i}}{\bar{t}}\nabla d_{i},$$

where $\omega_{i}$ are the two non-negative coefficients, which are used to control the amplification strength. Our method is executed on the multiple residual layers of the luminance channel $I_{L}$, rather than on the whole image. In addition, directly applying the reciprocal of *t* may cause the denominator to be close to zero, thereby producing artifacts. With these considerations, the mean value of *t* is utilized instead of *t* in Equation (13).

Once the enhanced gradients are computed, the final enhanced details can be produced by solving a Poisson equation on the amplified gradients, which is described as:

(14) $$d_{i}^{\prime }=\mathrm{Restore}\left(\nabla d_{i}^{\prime }\right).$$

According to the base layer *b* and reconstructed details $d_{i}^{\prime }$, the enhanced luminance channel $I_{L^{\prime }}$ can be expressed as follows:

(15) $$I_{L^{\prime }}=b_{L}+\sum_{i=1}^{k-1}d_{i}^{\prime }.$$

**Figure 3:**
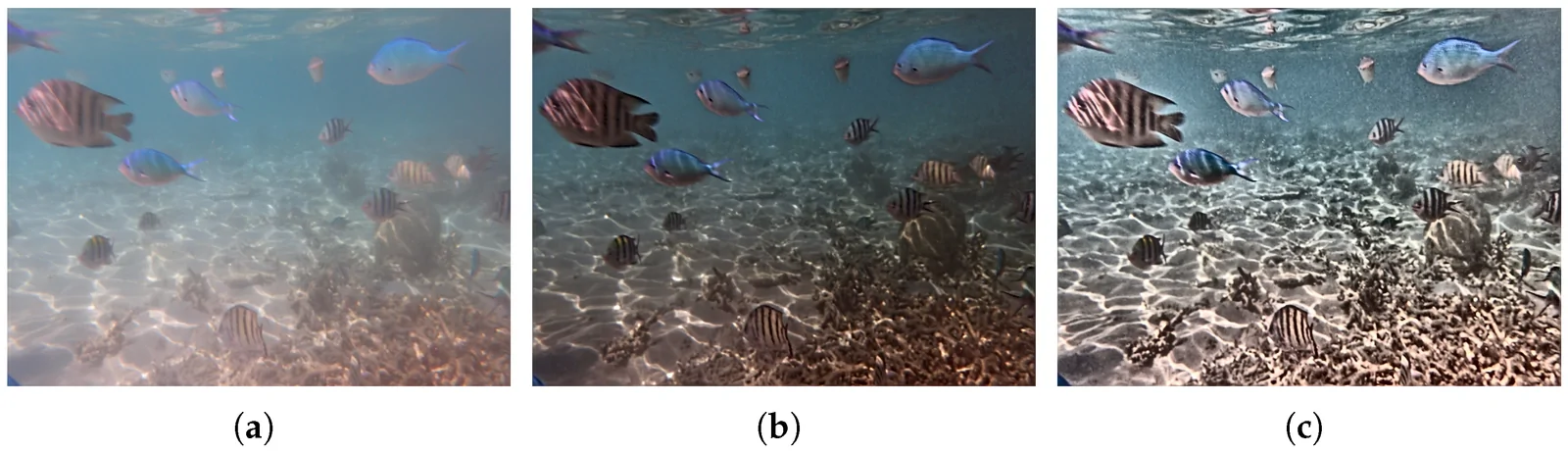
Comparative results of different methods. From (**a**–**c**): the raw image, the enhanced result without brightness adjustment, and our full method. As can be observed, our method can revise the global illumination of the entire image, revealing more texture in both foreground and background regions.

As can be seen from Figure 3, the detail enhancement of the gradient domain can remove a mass of haze and significantly improve the contrast and clarity. However, the light is strongly attenuated by the water body, and the brightness of underwater images is generally low. Thus, we use adaptive histogram equalization to stretch the global brightness of underwater images, which can be written as follows:

(16) $$I_{L^{\prime }}^{E}=\mathrm{AHE}\left(I_{L^{\prime }}\right).$$

### 3.3. Scene Transmission-Driven Color Restoration

**Figure 4:**
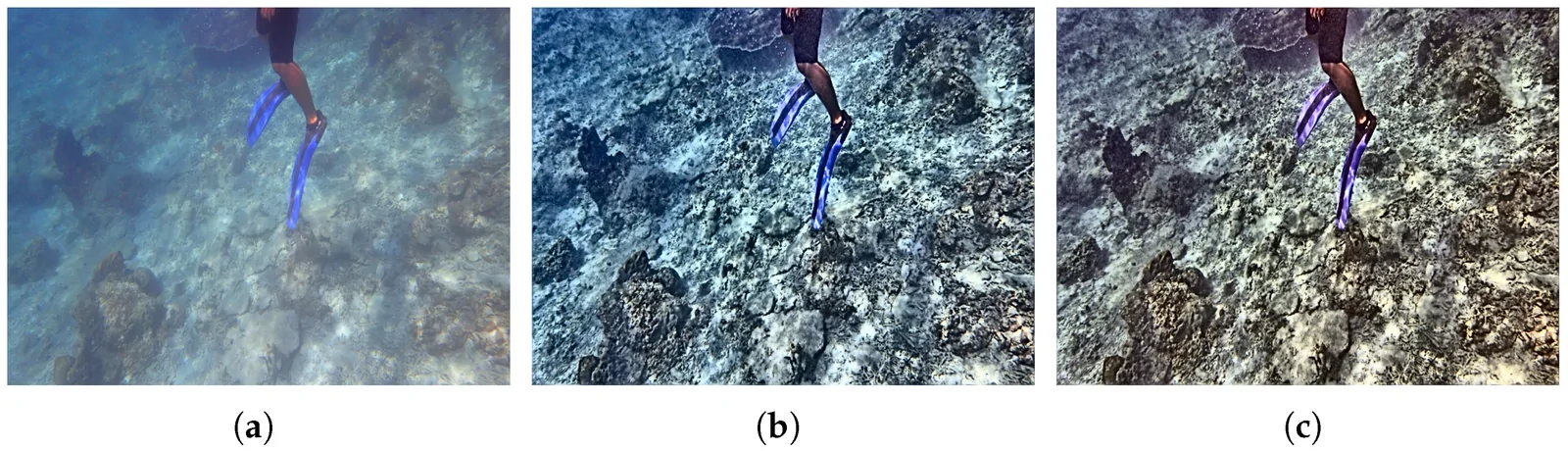
Comparative results of different methods. From (**a**–**c**): the raw image, the enhanced result of multiple-enhanced-layers fusion, and our full method. As can be observed, our method can produce a well-corrected result.

As shown in Figure 4, raw underwater images are characterized by a hazy appearance and bluish tone, generating low contrast, blurry details, and color casts. Although we enhance the contrast and the details based on the operations of Section 3.2, the color casts are not addressed well. As we know, wavelength-dependent attenuation is the main reason for causing color casts, and the more severe the attenuation is, the more obvious the color deviation will be. Meanwhile, according to underwater imaging model, lower transmission values indicate stronger contamination by the backscatter light component, resulting in perceptible color shifts from the true chromaticity. Such shifts manifest in color-opponent spaces, specifically, as global displacements in the chrominance channels (e.g., the *a* and *b* channels in the CIE Lab space). Consequently, the statistical distribution of transmission naturally provides a reliable basis for estimating the global color cast intensity.

The implementation uses the MATLAB rgb2lab and lab2rgb transformations with the default RGB color space and D65 reference white. Let *C* denote a normalized RGB component, where C∈{R,G,B}. Set δ=6/29 and $\left(X_{n},Y_{n},Z_{n}\right)=\left(0.9504,1.0000,1.0888\right)$ for the D65 reference white. The forward RGB-to-Lab conversion used in this work is:

(17) $$\begin{array}{cc} C_{\mathrm{lin}} & =\left\{\begin{array}{cc} C/12.92, & C\leq 0.04045,\\ {\left((C+0.055)/1.055\right)}^{2.4}, & C>0.04045, \end{array}\right.\\ \left[\begin{array}{c} X\\ Y\\ Z \end{array}\right] & =\left[\begin{array}{ccc} 0.4124564 & 0.3575761 & 0.1804375\\ 0.2126729 & 0.7151522 & 0.0721750\\ 0.0193339 & 0.1191920 & 0.9503041 \end{array}\right]\left[\begin{array}{c} R_{\mathrm{lin}}\\ G_{\mathrm{lin}}\\ B_{\mathrm{lin}} \end{array}\right],\\ L^{*} & =116f(Y/Y_{n})-16,\\ a^{*} & =500\left[f\left(X/X_{n}\right)-f\left(Y/Y_{n}\right)\right],\\ b^{*} & =200\left[f\left(Y/Y_{n}\right)-f\left(Z/Z_{n}\right)\right],\\ f(q) & =\left\{\begin{array}{cc} q^{1/3}, & q>\delta^{3},\\ q/(3\delta^{2})+4/29, & q\leq \delta^{3}. \end{array}\right. \end{array}$$

The inverse Lab-to-RGB conversion is:

(18) $$\begin{array}{cc} f_{y} & =\left(L^{*}+16\right)/116,\;f_{x}=f_{y}+a^{*}/500,\;f_{z}=f_{y}-b^{*}/200,\\ X & =X_{n}f^{-1}\left(f_{x}\right),\;Y=Y_{n}f^{-1}\left(f_{y}\right),\;Z=Z_{n}f^{-1}\left(f_{z}\right),\\ \left[\begin{array}{c} R_{\mathrm{lin}}\\ G_{\mathrm{lin}}\\ B_{\mathrm{lin}} \end{array}\right] & ={\left[\begin{array}{ccc} 0.4124564 & 0.3575761 & 0.1804375\\ 0.2126729 & 0.7151522 & 0.0721750\\ 0.0193339 & 0.1191920 & 0.9503041 \end{array}\right]}^{-1}\left[\begin{array}{c} X\\ Y\\ Z \end{array}\right],\\ f^{-1}\left(q\right) & =\left\{\begin{array}{cc} q^{3}, & q>\delta ,\\ 3\delta^{2}\left(q-4/29\right), & q\leq \delta , \end{array}\right.\\ C & =\left\{\begin{array}{cc} 12.92C_{\mathrm{lin}}, & C_{\mathrm{lin}}\leq 0.0031308,\\ 1.055C_{\mathrm{lin}}^{1/2.4}-0.055, & C_{\mathrm{lin}}>0.0031308. \end{array}\right. \end{array}$$

The average value μ of the transmission represents the global average haze or scattering intensity, while the standard deviation σ quantifies the spatial dispersion of transmission, i.e., the heterogeneity of depth distribution. Together, they form a two-dimensional feature tuple (μ,σ) that characterizes the degradation state, which can be expressed as:

(19) $$\mu =\frac{1}{N}\sum_{i=1}^{N}t_{i},$$

(20) $$\sigma =\sqrt{\frac{1}{N}\sum_{i=1}^{N}{\left(t_{i}-\mu \right)}^{2}},$$

where *N* is the total number of pixels in the transmission. To map the statistical features to an appropriate color-correction magnitude, we introduce a saturating sigmoidal mapping function that yields the correction strength factor *r*:

(21) $$r=t_{\min}+\frac{2\left(t_{\max}-t_{\min}\right)}{1+e^{-(\mu -\sigma )}},$$

where $t_{\min}$ and $t_{\max}$ are predefined lower and upper bounds that constrain the correction magnitude within a reasonable range. This mapping is motivated by three key considerations:

Monotonicity: *r* increases monotonically with (μ−σ), implying that lower average transmission and greater transmission variability jointly demand stronger correction.

Nonlinear saturation: The sigmoid form ensures that the correction strength saturates under extreme degradation conditions, preventing oscillation or over-correction caused by transmission estimation noise.

Variance-aware response: The inclusion of σ enables differential treatment of images sharing the same mean transmission but differing in variance—larger variance, which often indicates abrupt depth changes and localized color casts, triggers a stronger global compensation.

From a perceptual viewpoint, the decorrelation property of the Lab space ensures that the correction process solely modifies chrominance information while preserving the original luminance contrast and textural details. Thus, according to the correction factor *r*, the compensated *a* and *b* channels can be written as:

(22) $$\left\{\begin{array}{c} a^{\prime }\left(x,y\right)=a\left(x,y\right)-r\cdot \bar{a},\\ b^{\prime }\left(x,y\right)=b\left(x,y\right)-r\cdot \bar{b}. \end{array}\right.$$

where $\bar{a}$ and $\bar{b}$ denote the corrected *a* and *b* channels, and $\bar{a}$ and $\bar{b}$ are the mean values of the *a* and *b* channels. Once the color casts have been corrected by Equation (22), we can produce the final enhanced image by fusing $I_{L^{\prime }}^{E}$, $a^{\prime }$ and $b^{\prime }$. As shown in Figure 4c, our method can significantly remove the unwanted bluish tone and restore the natural appearance.

For reproducibility, all parameters and fixed implementation settings are collected in Table 1. The fixed parameters were selected empirically to balance enhancement strength, detail preservation and numerical stability. The quantities marked as adaptive are calculated separately from each input image and require no manual tuning.

## 4. Experiment Results and Discussion

This section first conducts an ablation study to evaluate the effectiveness of each configuration, and then provides comparative results between our restoration and other methods on three underwater image datasets. The compared methods consist of HFM [31], DAUT [32], WWPF [8], UShape [33], OGO-ULAP [34], PCFB [35], SDGE [36], LANet [37], PUIENet [38], DeepWav [39]. Except for qualitative comparisons, we also use five quantitative evaluation metrics to objectively test the enhancement results of these methods: *e* [40], $\bar{r}$ [40], Blur [41], UIQM [42], and DFAD [43]. Higher values of *e*, $\bar{r}$, and UIQM show superior contrast, sufficient details, and natural colors regarding the enhancement results, while lower values of Blur and DFAD demonstrate good clarity. Finally, we show the scatter diagram of different methods to further validate the color restoration ability. We also conduct a comparative experiment in terms of the salient detection and generalization ability on outdoor hazy images. Our experiments are tested on a 64-bit PC using MATLAB R2021b, interfaced with AMD Ryzen 9 5900X CPU@3.70 GHz, 32 GB RAM (Advanced Micro Devices, Inc., Santa Clara, CA, USA).

### 4.1. Evaluation Metrics

The five no-reference metrics used in this work are briefly defined below. Let $I_{o}$ and $I_{r}$ denote the input and restored images, respectively. The *e* and $\bar{r}$ are defined as:

(23) $$e=\frac{n_{r}-n_{o}}{n_{o}},\;\bar{r}=\exp\left(\frac{1}{n_{r}}\sum_{p\in P_{r}}\log r_{p}\right),$$

where $n_{o}$ and $n_{r}$ represent the numbers of visible edges in $I_{o}$ and $I_{r}$, respectively. $P_{r}$ denotes the set of visible edges in $I_{r}$, and $r_{p}$ is the gradient ratio at edge *p*. Here, *e* measures the relative number of newly visible edges after restoration, while $\bar{r}$ represents the geometric mean of the visibility-level gain over the restored visible edges. Larger values of both metrics indicate stronger contrast restoration.

The Blur metric measures the perceptual blur in the luminance image *F*. Let $B_{d}$ be the image obtained by applying a one-dimensional low-pass filter along direction d∈{ver,hor}, $D_{F,d}$ and $D_{B,d}$ be the absolute differences of neighboring pixels in *F* and $B_{d}$, respectively, and $s_{F,d}$ and $s_{V,d}$ be the sums of $D_{F,d}$ and $V_{d}=\max\left(0,D_{F,d}-D_{B,d}\right)$. It is defined as:

(24) $$\mathrm{Blur}=\max_{d\in \{\mathrm{ver},\mathrm{hor}\}}\left(\frac{s_{F,d}-s_{V,d}}{s_{F,d}}\right).$$

This score ranges from 0 to 1; a lower value indicates a sharper image.

UIQM is a composite underwater image-quality measure that jointly assesses colorfulness, sharpness, and contrast:

(25) UIQM=0.0282UICM+0.2953UISM+3.5753UIConM,

where

(26) $$\begin{array}{cc} \mathrm{UICM} & =-0.0268\sqrt{\mu_{\alpha ,RG}^{2}+\mu_{\alpha ,YB}^{2}}+0.1586\sqrt{\sigma_{\alpha ,RG}^{2}+\sigma_{\alpha ,YB}^{2}},\\ \mathrm{UISM} & =\sum_{c\in \{R,G,B\}}\lambda_{c}\;\mathrm{EME}\left(\mathrm{Sobel}(I_{c})\right),\\ \mathrm{UIConM} & =-\frac{1}{K_{1}K_{2}}\sum_{k=1}^{K_{1}}\sum_{l=1}^{K_{2}}C_{kl}\log C_{kl},\;C_{kl}=\frac{Y_{\max,kl}-Y_{\min,kl}}{Y_{\max,kl}+Y_{\min,kl}}. \end{array}$$

In these expressions, RG=R−G and $YB=\frac{1}{2}\left(R+G\right)-B$; $\mu_{\alpha ,\cdot }$ and $\sigma_{\alpha ,\cdot }$ denote the asymmetric α-trimmed mean and standard deviation; EME(·) measures edge-based enhancement; $\lambda_{R}=0.299$, $\lambda_{G}=0.587$, and $\lambda_{B}=0.114$; and $Y_{\max,kl}$ and $Y_{\min,kl}$ are the maximum and minimum luminance values in the (k,l)-th image block. A larger UIQM indicates better overall underwater image quality.

Finally, the DFAD values are obtained using the perceptual fog-density predictor FADE [43]. Let $D_{f}$ be the Mahalanobis-like distance between the fog-aware natural-scene-statistics (NSSs) model fitted to the test image and the nominal fog-free model, and let $D_{ff}$ be its distance to the nominal foggy model. The DFAD is defined as:

(27) $$\mathrm{DFAD}=\frac{D_{f}}{D_{ff}+1}.$$

A lower DFAD value represents lower perceived fog density and, therefore, a better dehazing performance.

### 4.2. Ablation Study

To assess the restoration performance of different configurations in our method, we use an ablation analysis on the UCCS [44], UIQS [44], and UIEB [21] datasets, which contains the following configurations: (a) our method without adaptive histogram equalization (-w/o AHE); (b) our method without color restoration (-w/o CR); (c) our method without detail enhancement (-w/o DE); (d) our method without saturation adjustment (-w/o SA); (e) our full method.

**Figure 5:**
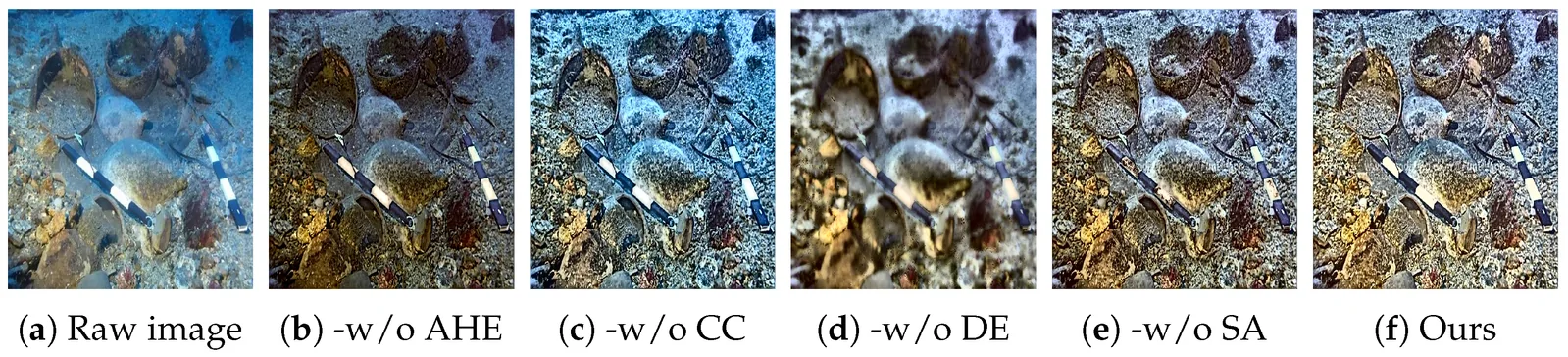
Comparative results using different configurations. As can be seen, our full method is effective for correcting the distorted color and the low contrast and brightness.

Figure 5 shows the visual enhancement result of each configuration, producing the following analysis: (1) -w/o AHE removes the haze and enhances the contrast, but the overall brightness is low, hiding some important details. (2) Compared with -w/o AHE, -w/o CC obviously improves the global brightness while preserving the contrast and the details. However, the unwanted bluish tone is still found in the result. (3) -w/o DE, although it works well in correcting the colors, cannot revise the clarity and increase the details. (4) -w/o SA yields local overexposure and amplified noise, and some regions suffer unnatural excessive color saturation with unbalanced visual performance. (5) Our full proposed method achieves state-of-the-art performance in haze removal, color correction, and fine detail restoration.

Table 2 reports the quantitative values of *e*, $\bar{r}$, and Blur on our tested datasets. As shown, our method obtains the best $\bar{r}$ and Blur values on the UCCS and UIQS datasets, especially for the $\bar{r}$ value; our method increases by at least 5.80% compared with the second-best method. Meanwhile, the average $\bar{r}$ value of our method increases by 10.83% on the UIEB. Although -w/o CC generates the best *e* values, the visual result is not good. Thus, based on the comprehensive analysis of qualitative and quantitative results, it can be proven that each core module positively affects our method.

### 4.3. Color Restoration Analysis on UCCS

**Figure 6:**
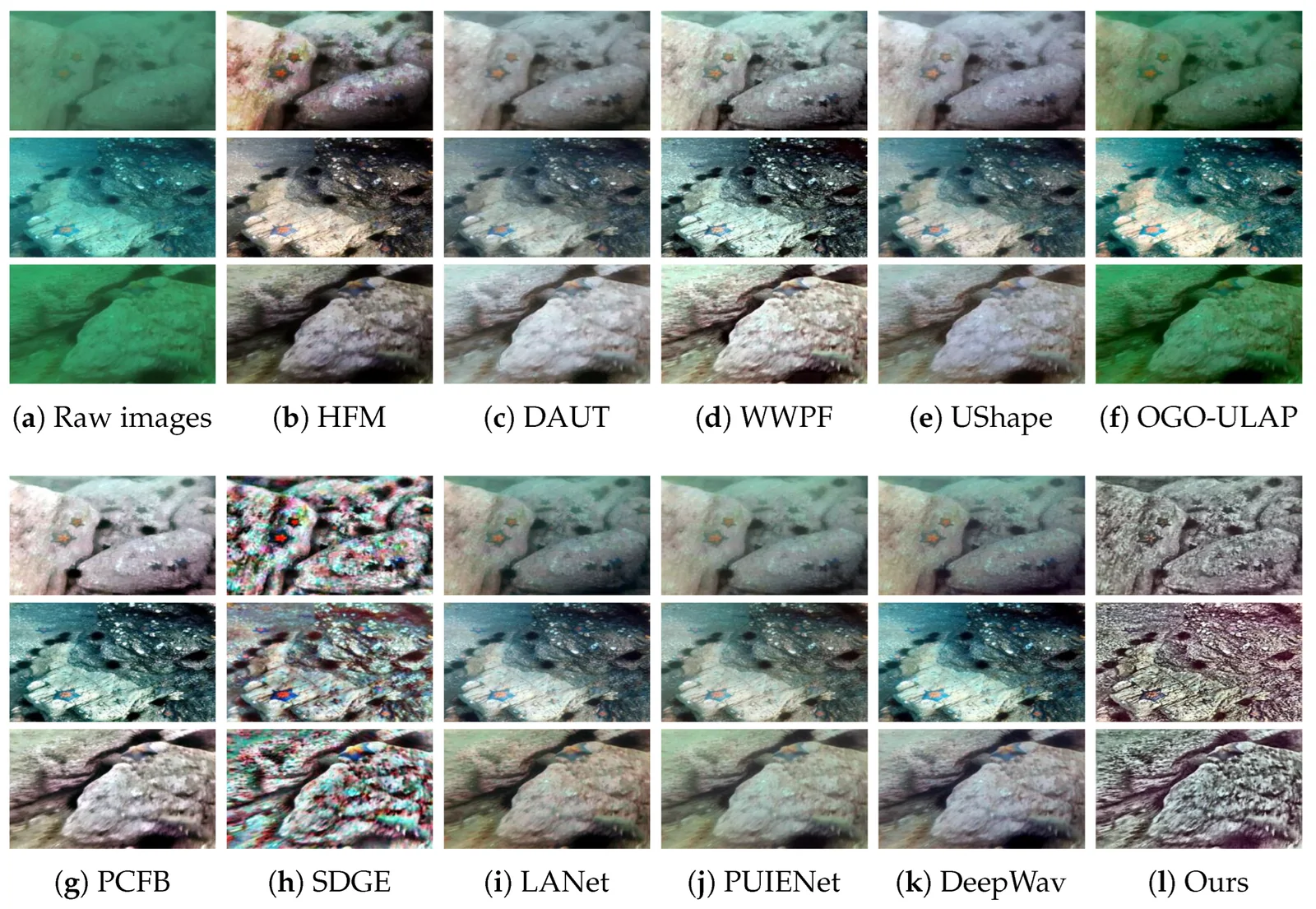
Enhancement results of different methods on the UCCS. As can be found, our method completely eliminates the color bias and restores the natural appearance.

Qualitative Comparisons: To demonstrate the color restoration performance of different enhancement methods, we conduct a comparative experiment on the UCCS dataset. As can be seen from Figure 6, the three raw images are characterized by blue-greenish haze, bluish haze, and greenish haze, respectively. For the blue-greenish underwater image, HFM revises the greenish appearance and removes the haze, improving the contrast, but the result shows local dark regions. DAUT and UShape produce similar enhancement results, and these results suffer from poor details and residual haze. WWPF obtains a more clear appearance compared with them. OGO-ULAP slightly enhances the brightness, but it fails to eliminate the color cast and the haze. PCFB removes the blue-greenish haze and produces a natural appearance; unfortunately, the details are insufficient. SDGE yields good enhancement results with high contrast and sufficient details. Meanwhile, SDGE also excessively amplifies reddish tones, such as that of the starfish. LANet and DeepWav reduce a large amount of haze and provide limited contrast enhancement results. In addition, their results contain the unwanted bluish appearance. PUIENet generates reasonable brightness, but it only removes the partial blue-greenish haze. Compared with them, our method effectively eliminates the haze and the color bias, offering the enhancement result with superior details, high contrast, and complete structure.

For the bluish underwater image, HFM corrects the color cast, but it creates highlighted foreground regions while producing dark background regions. The enhancement results of DAUT and UShape are similarly blurry. WWPF generates a clearer enhancement result; however, it also suffers from unbalanced illumination. OGO-ULAP produces some overexposed regions in the stones, losing the texture information. PCFB is less capable of correcting the blue bias than correcting the blue-greenish cast, and shows a residual bluish tone. SDGE obtains global reasonable brightness without any underexposed and overexposed regions. LANet and DeepWav contain obvious blue bias in the background regions, which demonstrates that they are bad for balancing the global colors. By contrast, our method achieves almost universal brightness and a colorful appearance.

For the greenish underwater image, the regions under the stones are dark in HFM’s result. OGO-ULAP is ineffective for revising the greenish tone, but it can more or less improve the details. The colors are pale in terms of the enhancement results of DAUT and UShape. LANet shows an additional brown bias. PUIENet reveals a slight greenish tone in the distant stones. Inversely, our method completely eliminates the green bias and restores the natural appearance. Meanwhile, the result does not introduce over-saturated and overexposed regions.

Quantitative Comparisons: Table 3 shows the average values of *e*, $\bar{r}$, Blur, UIQM, and DFAD metrics in terms of the enhancement results on the UCCS dataset. As shown, our method achieves the best values of *e*, $\bar{r}$, Blur, and DFAD. Compared with the second-best value, the average values of *e* and $\bar{r}$ significantly increase by 3.87% and 28.86%, respectively, which implies that our results have sufficient information, and the colorful and detailed fidelity of the restoration results is preferable. Meanwhile, the average Blur and DFAD values of our method obviously decline by 10.15% and 2.36%, respectively, demonstrating the clear appearance and high contrast of our enhancement results.

### 4.4. Comprehensive Comparisons of Visibility Improvement on UIQS

**Figure 7:**
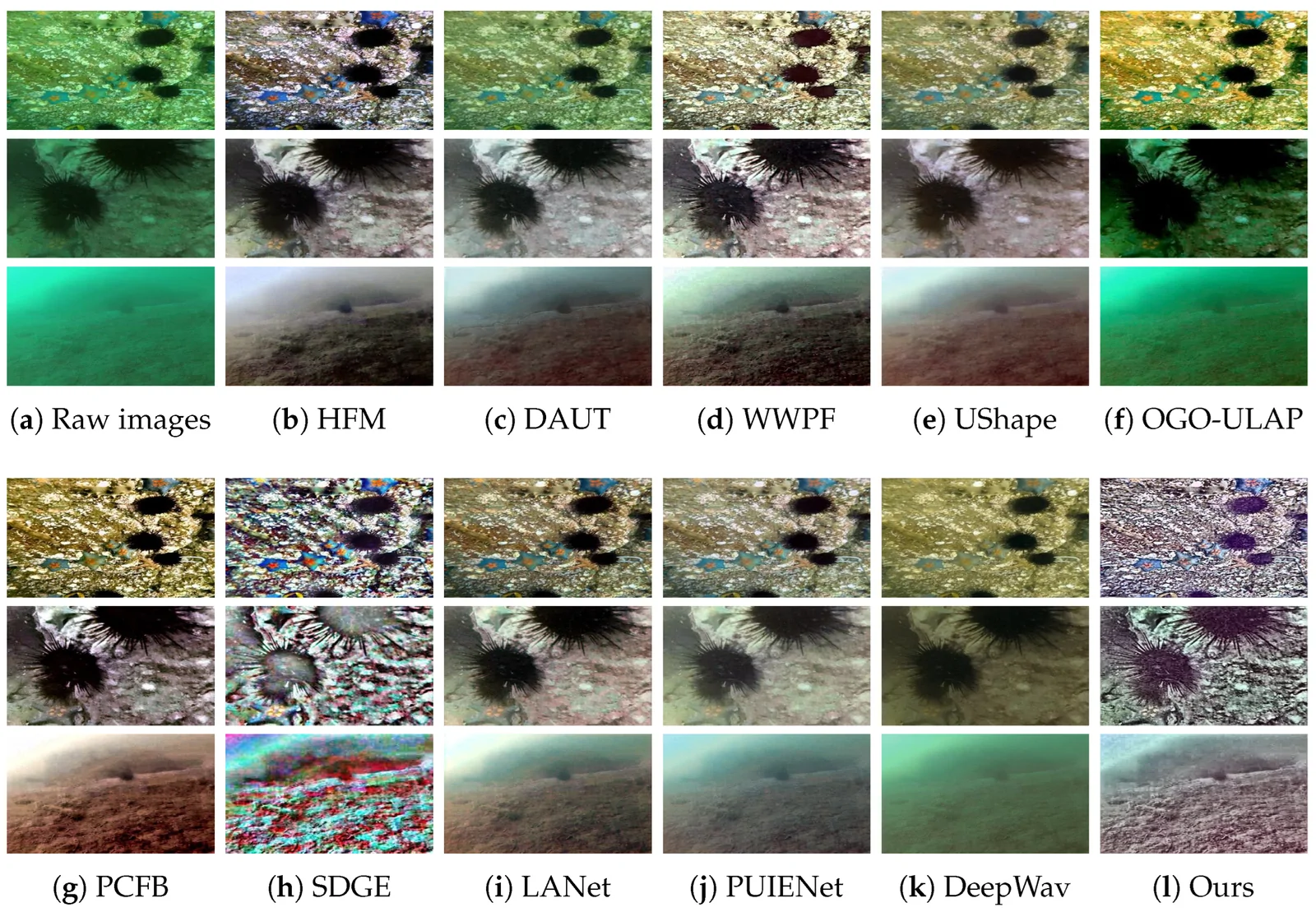
Enhancement results of different methods on the UIQS. As can be found, our method simultaneously realizes complete color bias correction, hierarchical haze suppression, and texture preservation across slight, moderate, and dense hazy scenes.

Qualitative Comparisons: To evaluate the enhancement ability of different methods on real-world underwater scenes, we use these methods to enhance the underwater images of UIQS. Figure 7 shows the detailed comparative results. As can be observed, three raw images suffer from slight, moderate, and dense haze, respectively, and also have diverse color casts. HFM achieves good enhancement results for slight and moderate hazy underwater images, but it shows residual haze for dense hazy images, providing low contrast. OGO-ULAP generates an unwanted yellowish tone and cannot eliminate the haze. DAUT and UShape over-smooth critical fine textures, such as sea urchin spines and sediment grains, and only correct the green bias in dense hazy scenes. WWPF causes some overexposed noises in the background regions; meanwhile, it also contains a mass of residual haze in the enhancement result. PCFB produces low edge sharpness and appears to have a reddish tone while eliminating severe green haze. Although SDGE significantly reduces the haze for all hazy scenes, the enhancement results are unnatural. For example, the shape of the sea urchin is severely distorted, and the entire image contains red noises, resulting in unrecognizable targets. DeepWav has weak dehazing ability and residual green haze remains across the background water. Compared with these methods, our method simultaneously realizes complete color bias correction, hierarchical haze suppression, and multi-scale texture preservation across slight, moderate, and dense hazy scenes, delivering optimal visual fidelity, structural clarity, and natural color reproduction.

Quantitative Comparisons: In addition, we also list the quantitative values of these methods on the UIQS dataset. It can be seen from Table 4 that our method achieves the best values of $\bar{r}$ and Blur, increasing and decreasing by 32.98% and 9.25% compared with the second-best method, respectively. Meanwhile, our method also obtains the second-best values of *e* and DFAD. These data show our method can provide helpful support for sharpening the details, enhancing the contrast, and improving the visibility quality.

### 4.5. Comprehensive Comparisons of Diverse Underwater Scenes on UIEB

**Figure 8:**
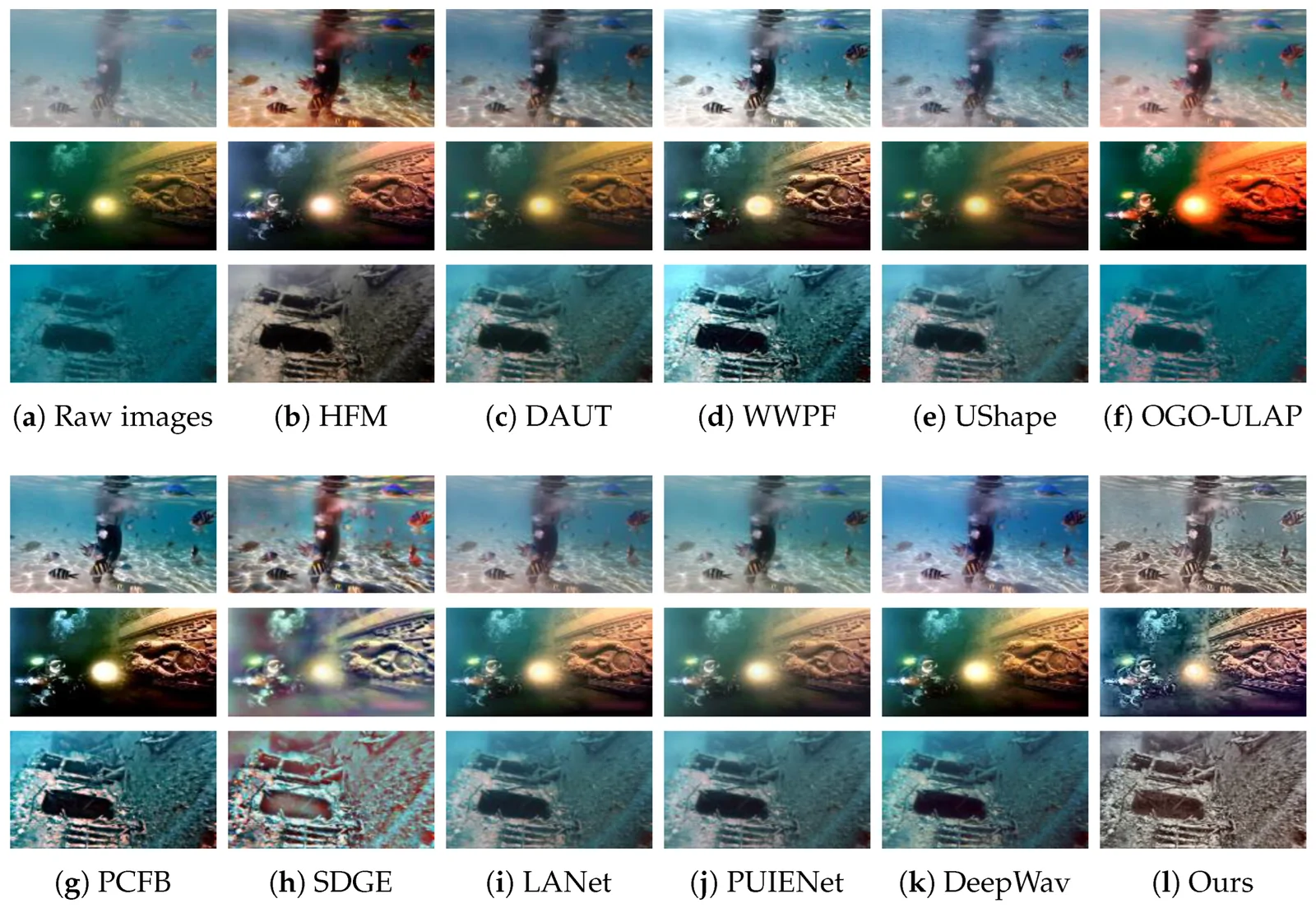
Enhancement results of different methods on the UIEB. As can be found, our method adaptively addresses the color casts of diverse underwater hazy scenes and reasonably suppresses uneven illumination without detail loss.

Qualitative Comparisons: To fully demonstrate the effectiveness and robustness of our method on diverse underwater scenes, we test the enhancement results of different methods on the UIEB dataset. As shown in Figure 8, the raw images are sourced from underwater diving and archaeological exploration scenarios. HFM mitigates underwater color bias and restores natural seabed tones, yet residual haze blurs fine textures with insufficient local contrast. DAUT and WWPF adopt excessive smoothing that blurs delicate structural features such as fish stripes and wreck carvings; they also introduce an incomplete color correction performance. WWPF corrects the global illumination of the entire images but erases tiny textures and blurs the boundaries of fish and wreck structures due to unreasonable brightness adjustment. Additionally, WWPF cannot revise the green bias on the seabed. OGO-ULAP fails to remove the haze and introduces artifact halos, producing poor enhancement results with low contrast, unnatural blue or orange tint, and blurry structure. Compared with it, PCFB shows superior enhancement performance in improving the structure and texture information. However, PCFB cannot remove the color casts of distant regions. SDGE generates severe rainbow chromatic noise in all underwater scenes, fully covering textures and causing irreversible visual distortion. LANet achieves partial haze removal but amplifies the effects of the illuminated source, losing fine details. PUIENet and DeepWav are more or less effective for adjusting the brightness and suppressing the haze. In contrast, our method adaptively addresses the color casts of diverse underwater hazy scenes and reasonably suppresses uneven illumination without detail loss. It fully retains micro-textures like fish stripes and wreck carvings, producing natural, high-fidelity visuals superior to these competitive methods.

Quantitative Comparisons: Similarly, we also use *e*, $\bar{r}$, Blur, UIQM, and DFAD to quantitatively evaluate the enhancement performance on the UIEB dataset. Table 5 shows the detailed values of these compared methods. As can be found, our method yields the best values in all evaluation metrics. More importantly, compared with the second-best method, the average *e*, $\bar{r}$, and UIQM values increase by 82.08%, 99.87%, and 1.98%, respectively, which means that our method can produce clear edges, sufficient contrast, and good color saturation. Meanwhile, the average Blur and DFAD values decrease by 10.22% and 9.18%, respectively, which implies that our method shows good performance in removing the haze. Therefore, based on the qualitative and quantitative comparisons, our method has stable effectiveness and robustness for diverse underwater image enhancement.

### 4.6. Color Histogram Analysis of Underwater Images

**Figure 9:**
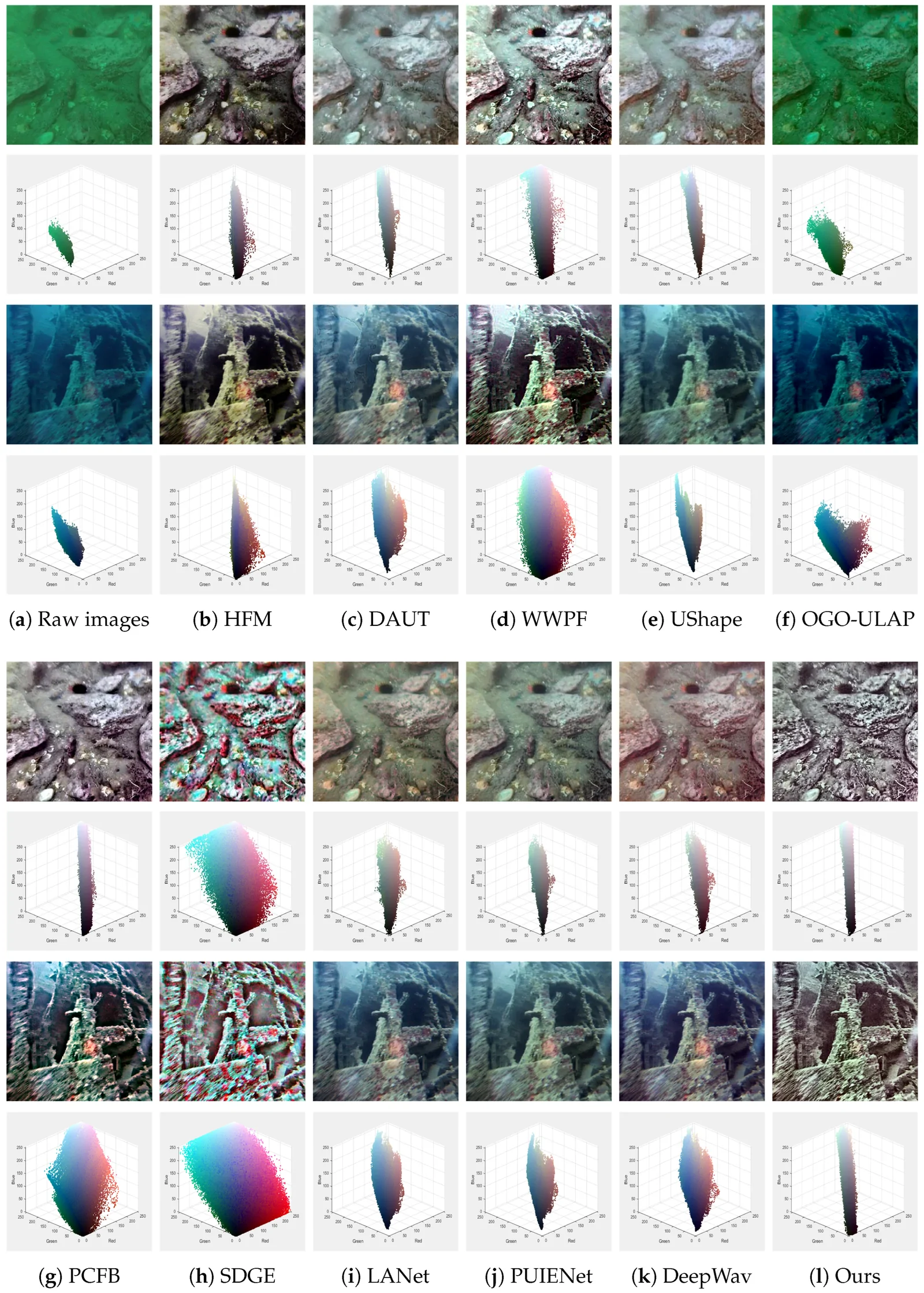
Enhancement results of different methods and the corresponding pixel distributions on RGB spatial coordinate systems. As can be observed, our method thoroughly disperses the green-blue concentrated RGB pixel clusters of raw underwater images, achieving balanced RGB distribution and optimal natural color restoration.

To further analyze the color restoration capability of different methods on underwater images, we assess the pixel distribution of the red, green, and blue color components based on RGB spatial coordinate systems. Figure 9 shows the pixel distribution on the RGB system in terms of the enhancement results of different methods. Raw underwater images skew heavily to green and blue channels. Their RGB scatter forms compact clusters with extremely insufficient red pixel distribution and narrow color gamut. HFM can work well in correcting the color distortion, which enables the pixel distribution pointing to the intersection of the three-dimensional coordinates. Since the enhancement contains some dark regions, most of the pixels are concentrated in the low-value range. WWPF significantly expands RGB distribution but excessively lifts red components, failing to achieve balanced three-channel coverage. DAUT and UShape also boost the dynamic range of red, green, and blue channels due to the haze suppression. However, the residual blue bias of background regions limits the balance of pixel distributions. SDGE produces severe color noise on images, whose RGB scatter distributes chaotically with numerous abnormal outliers across all three color axes. LANet and DeepWav produce more blue deviations in the enhancement results compared with PUIENet, generating a wider distribution of blue pixels. OGO-ULAP only eliminates a small amount of haze and cannot revise the green and blue bias, making weak stretching in scatter plots. Compared with them, our method thoroughly disperses the green-blue concentrated RGB pixel clusters of raw underwater images. It evenly stretches pixels across the full three-channel range without noisy outliers, achieving balanced RGB distribution and optimal natural color restoration, which strictly meets the famous gray-world assumption, revealing the effectiveness of our color correction.

### 4.7. Analysis of Detail Enhancement

**Figure 10:**
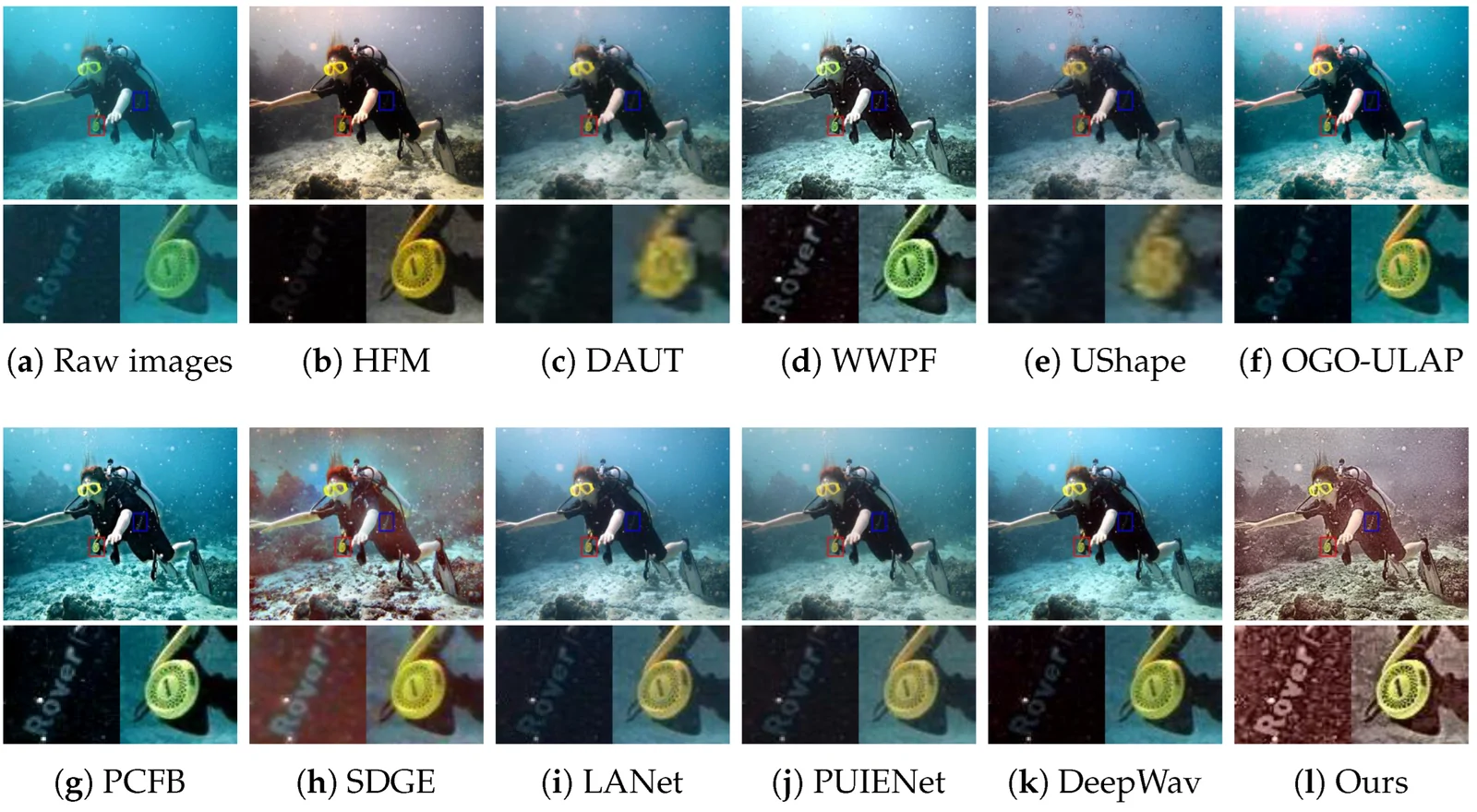
Visual comparisons of detail enhancement results using different methods. As can be seen, our method obviously shows clear text and object textures.

Since high-quality underwater images should contain superior texture details, we conduct a detail enhancement analysis of different methods. The detailed comparisons are shown in Figure 10. As can be observed in the enlarged patches, raw underwater image suffers from severe color cast, low contrast, and blurred fine-grained textures. HFM, DAUT, UShape and OGO-ULAP reduce the brightness of the text, making it almost impossible to see. and SDGE introduce obvious color shift and artifacts, bringing unnatural visual effects. Other methods recover limited local information, where subtle text and edge structures remain blurred even after enhancement. By contrast, our method achieves promising haze removal and color correction, while well-preserving high-frequency textures. Fine details in magnified regions are distinctly enhanced without over-smoothing, demonstrating superior detail enhancement performance.

### 4.8. Analysis of Image Salient Detection

**Figure 11:**
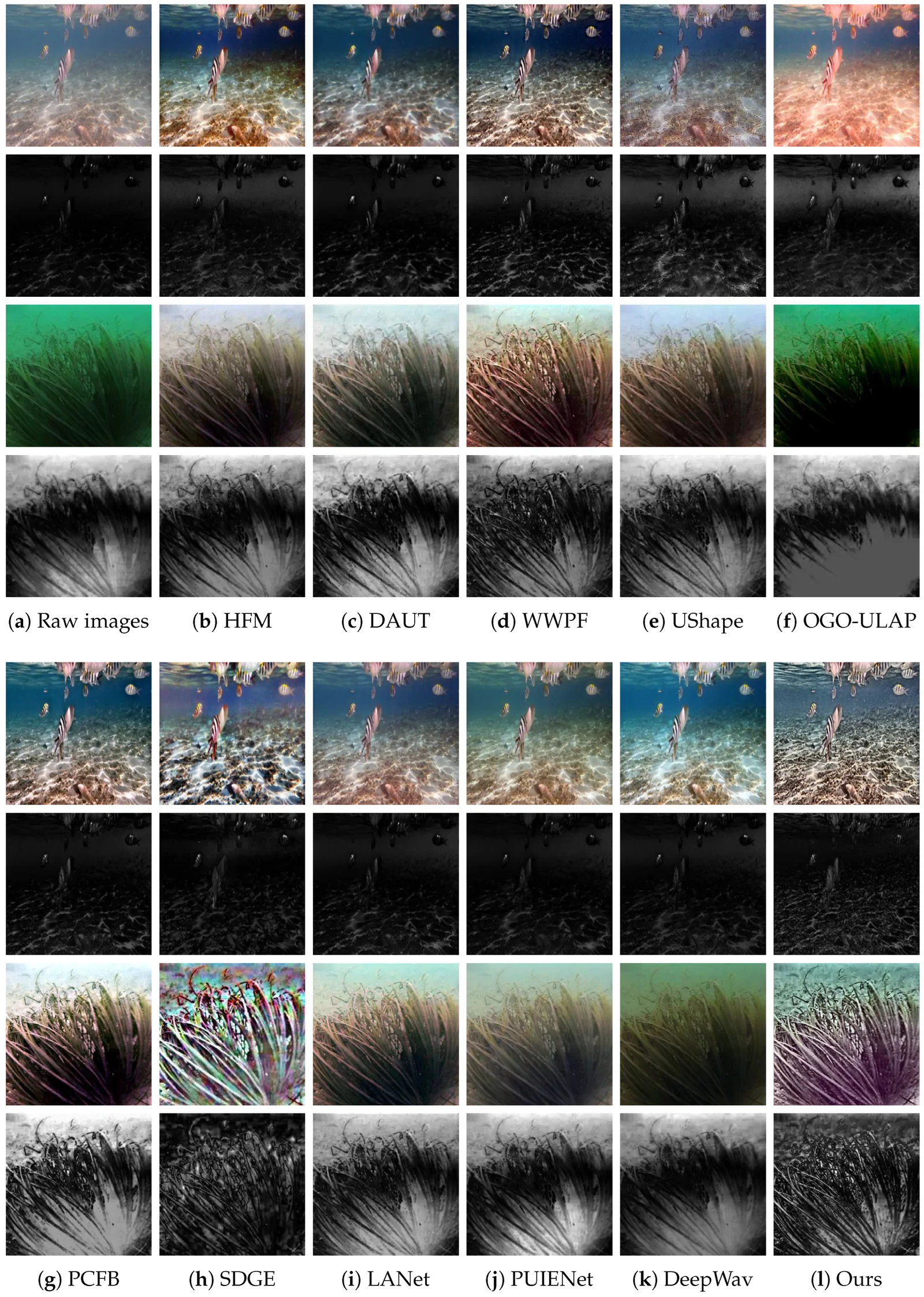
Enhancement results of different methods and the corresponding salient detection results. As can be observed, our saliency maps produce complete and clear foreground highlights, suppress background interference, and accurately outline delicate target textures.

Salient object detection is an important vision task and is highly desired for high-quality underwater images. Thus, we analyze the salient detection results of different enhancement images produced by the comparative methods. Figure 11 shows the corresponding detection results. Raw underwater images suffer from severe color casts and low contrast. Their saliency maps show dim, fragmented foreground responses, mixed with heavy background noise and blurry target boundaries. HFM eliminates the color casts and improves overall contrast. Its saliency maps enhance the texture information but fail to detect the distant object. Compared with it, WWPF provides good enhancement results with high contrast and natural colors, thereby producing fine detection results, such as the fish pattern. OGO-ULAP introduces an unwanted reddish tone and cannot correct the green bias. Meanwhile, it is ineffective for reducing the haze. Therefore, the salient detection results are the worst. SDGE introduces severe red noise in the enhancement results, and the saliency maps are filled with messy false contours, failing to distinguish real foreground targets and producing massive invalid background salient responses. Other methods yield limited enhancement results. Their saliency maps contain some shortcomings: unclear texture, incomplete structure, et al. By contrast, our method obviously removes underwater color distortion and restores natural balanced hues without extra noise. In addition, our saliency maps produce complete and clear foreground highlights, suppress background interference, and accurately outline delicate target textures.

### 4.9. Comparisons of Running Time

We further conduct comparisons of the running time of different methods. HFM, WWPF, OGO-ULAP, PCFB, SDGE and our methods are tested on a 64-bit PC using MATLAB R2021b, which interfaced with AMD Ryzen 9 5900X CPU@3.70 GHz, 32 GB RAM. Deep learning-based methods are tested on an Ubuntu 20.04 PC with a PyTorch 1.11.0, Python 3.8 environment, equipped with an NVIDIA RTX 4090 (24 GB) GPU and 20 vCPU (Intel(R) Xeon(R) Platinum 8470Q) CPU, and CUDA 11.3. Table 6 shows the running time of different methods on three images with size 256×256, 640×480, and 1800×1295. It should be noted that DAUT and UShape can only process the image with a size 256 × 256, and PUIE-Net cannot process 1800×1295 images due to the limitation of the network structure. As can be seen, PUIENet achieves the best inference speed on 256×256 and 640×480 images. Although our method consumes more time, it yields favorable visual and quantitative enhancement performance. Meanwhile, we will develop a fast optimization strategy to accelerate the proposed method in our future works.

### 4.10. Generalization Capacity of Our Method

**Figure 12:**
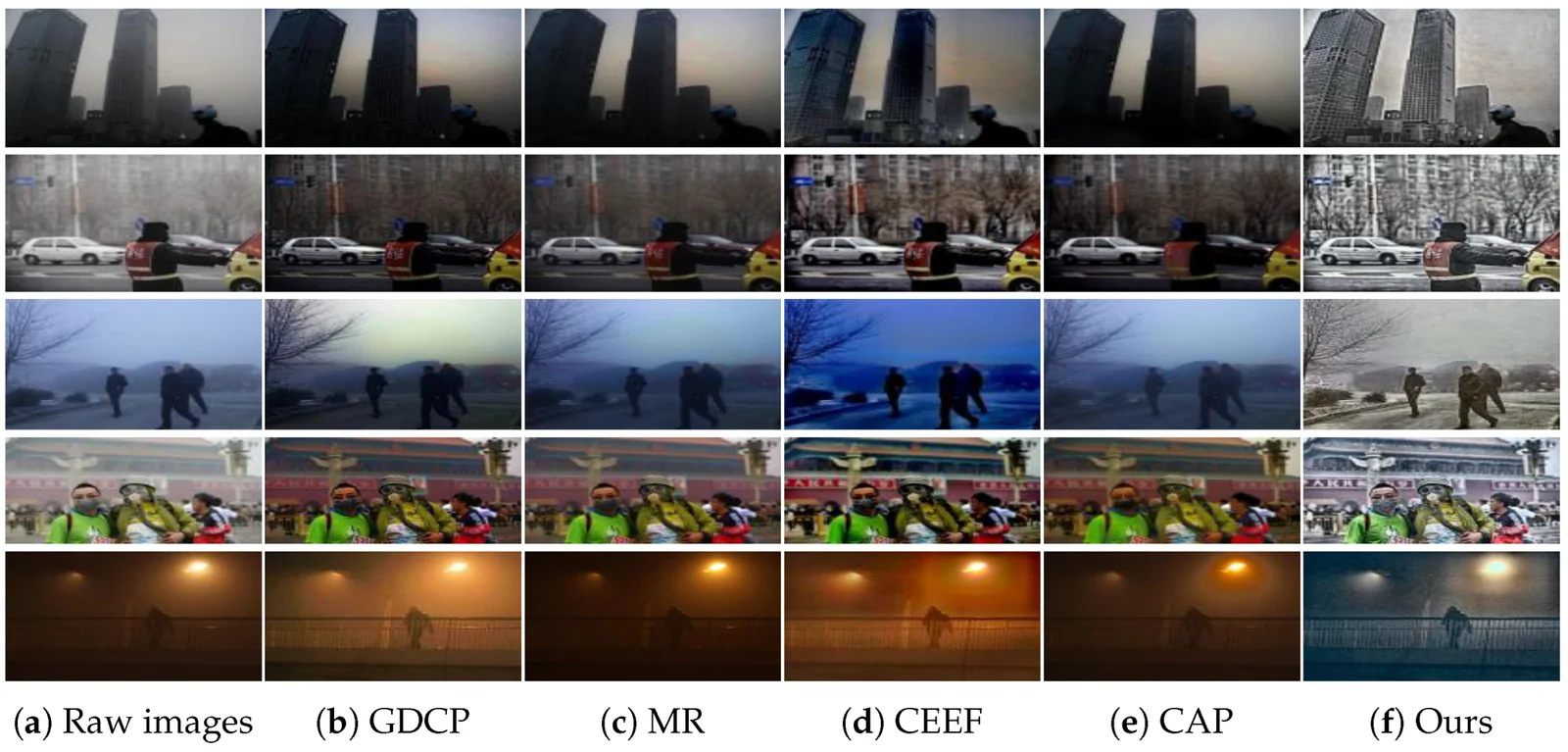
Generalization performance on the outdoor hazy images. Our method yields the desired visibility enhancement results, producing superior enhancement results with good illumination, high contrast, and sufficient details.

Our method yields good enhancement performance for underwater images. This section uses our method to restore outdoor hazy images, and the raw images are captured from tall buildings, urban streets, crowd scenic spots, and night-time hazy scenes. The corresponding enhancement results are listed in Figure 12. It can be observed that GDCP [45] can remove the bluish tone of background regions and enhance the brightness of the night-time hazy environment. Unfortunately, it causes some local dark regions. MR [46] and CAP [47] produce the similar enhancement appearance. However, they fail to correct the color casts. CEEF [48] shows clear appearance and good brightness in the enhancement results, but the bluish and yellowish tones can not be removed. Compared with these methods, our method shows a certain of enhancement advantages. Concretely, for the tall building scene, our method eliminates dense haze, restores clear window textures, and balances contrast well without color distortion or overexposure. For urban street and crowd scenic spot scenes, our method restores complete outlines of distant buildings and details of roads, cars, and pedestrians. Meanwhile, the texts of the distant regions are clear in our results. For the night-time hazy scene, our method eliminates outdoor haze under dim night lighting, balances dark brightness, and restores clear human and railing outlines without darkness collapse or color distortion. Meanwhile, Table 7 shows the average *e*, $\bar{r}$ and DFAD values of different methods on the Figure 12. As shown, consistent with the subjective performance, our method achieves the best quantitative results across all evaluation metrics, which shows a good generalization performance.

### 4.11. Failure Cases

**Figure 13:**
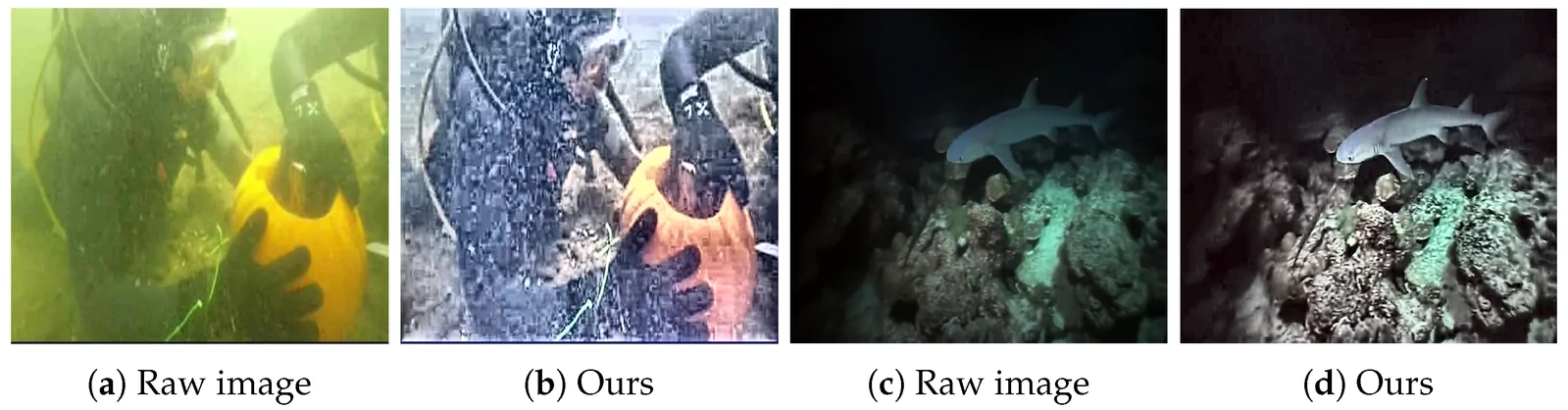
Generalization performance on the challenging scenes. Our method yields the desired visibility enhancement results, producing superior enhancement results with good illumination, high contrast, and sufficient details.

Although our method achieves good enhancement results in most underwater scenes, it shows failure cases when applied to challenging scenes. As displayed in Figure 13a,b, the raw image contains a yellowish haze and a large amounts of suspend particles. Our method can correct the color casts, remove the haze and enhance the details. However, it also over-amplifies the effect of these suspended particles, creating unwanted noise. Meanwhile, as depicted in Figure 13c,d, the raw image is characterized by non-uniformed illumination. Our method can more or less improve the local brightness and the clarity, but fails to enhance the global illumination, especially for the background regions. The primary reason is that our proposed method relies on the atmospheric scattering model, and this model does not take into account the suspended noises and non-uniform illumination. In future work, we will construct a physical model that unifies the visibility of suspended noises and illumination and deal with these challenging cases.

## 5. Conclusions

This paper introduces a simple yet effective underwater image enhancement method, which consists of three main modules: a pixel-based transmission estimation, a multiple-enhanced-layers fusion, and a transmission-driven color restoration. First, we use a mapping relationship between the transmission, the saturation, and the brightness to estimate the transmission, where the saturation is revised by an adjustment parameter, avoiding the under- and overestimation. Then, we use the estimated transmission to enhance the gradients of multiple detail layers, improving the details of the enhancement results. In addition, we also apply an adaptive histogram equalization strategy to expand the dynamic range, achieving global brightness revision. Finally, we fully take into account the high correlation between the transmission and color degradation, and design a compensation strategy to dynamically compensate for the color attenuation of diverse underwater scenes. Qualitative and quantitative results on three underwater image datasets demonstrate the effectiveness and robustness of our method. Meanwhile, extensive experiments also further suggest that our method can also provide beneficial support for salient detection and outdoor hazy image enhancement.

## Figures and Tables

**Table 1:** Summary of the parameters and implementation settings used in the proposed method.

Module	Parameter or Setting	Value	Determination and Role
PTE	Saturation gain α	0.1	Fixed empirical value controlling the saturation compensation in Equation (9).
MELF	Number of representations *k*	3	Fixed structural choice determining the two residual detail layers through the index range i=1,…,k−1 in Equation (12).
MELF	WLS regularization $\phi_{1},\phi_{2}$	0.1,1.0	Fixed empirical scales used to generate the mildly and strongly smoothed luminance representations in Equation (11).
MELF	Detail weights $\omega_{1},\omega_{2}$	2,4	Fixed empirical gains in Equation (13); the lower gain protects fine-scale details from noise amplification, while the higher gain strengthens coarser structures.
TCR	Sigmoid bounds $t_{\min},t_{\max}$	min(t),max(t)	Image-adaptive lower and upper transmission statistics in Equation (21), used to bound the correction strength *r*.

**Table 2:** Quantitative *e*, $\bar{r}$ and Blur values using different modules on the UCCS, UIQS and UIEB datasets. The best result is shown in red.

Datasets	Evaluations	-w/oAHE	-w/oCC	-w/oDE	-w/oSA	Ours
UCCS	*e*	7.352	37.306	9.449	33.797	33.740
	$\bar{r}$	2.544	7.246	2.842	7.274	7.696
	Blur	0.299	0.293	0.396	0.300	0.292
UIQS	*e*	6.158	50.994	9.702	42.422	41.914
	$\bar{r}$	2.489	6.904	2.725	6.914	7.442
	Blur	0.304	0.296	0.408	0.303	0.294
UIEB	*e*	2.252	8.182	3.394	7.893	8.068
	$\bar{r}$	2.181	5.574	2.208	5.436	6.178
	Blur	0.244	0.236	0.347	0.242	0.237

**Table 3:** Average quantitative results of different methods on the UCCS dataset. The best result is shown in red and the second best result is shown in blue.

Method	HFM	DAUT	WWPF	UShape	OGO-ULAP	PCFB	SDGE	LANet	PUIENet	DeepWav	Ours
*e*	25.969	7.679	25.137	9.611	5.318	20.201	32.481	9.247	5.910	11.808	33.740
$\bar{r}$	2.920	2.528	4.475	2.414	1.729	4.015	5.972	2.249	2.042	2.416	7.696
Blur	0.356	0.441	0.325	0.404	0.333	0.358	0.415	0.358	0.362	0.334	0.292
UIQM	1.403	1.102	1.458	1.147	1.211	1.410	1.541	1.216	1.135	1.267	1.455
DFAD	0.389	0.722	0.323	0.637	0.398	0.390	0.254	0.595	0.755	0.522	0.248

**Table 4:** Average quantitative results of different methods on the UIQS dataset. The best result is shown in red and the second best result is shown in blue.

Method	HFM	DAUT	WWPF	UShape	OGO-ULAP	PCFB	SDGE	LANet	PUIENet	DeepWav	Ours
*e*	33.849	8.993	43.256	6.240	4.578	34.445	55.086	9.830	6.070	0.482	41.914
$\bar{r}$	2.763	2.184	4.219	2.109	1.711	3.727	5.596	2.235	1.955	1.089	7.442
Blur	0.356	0.437	0.326	0.408	0.335	0.357	0.413	0.359	0.362	0.324	0.294
UIQM	1.403	1.132	1.469	1.155	1.260	1.419	1.527	1.252	1.115	0.910	1.453
DFAD	0.408	0.698	0.329	0.679	0.359	0.384	0.242	0.583	0.753	1.012	0.264

**Table 5:** Average quantitative results of different methods on the UIEB dataset. The best result is shown in red and the second best result is shown in blue.

Method	HFM	DAUT	WWPF	UShape	OGO-ULAP	PCFB	SDGE	LANet	PUIENet	DeepWav	Ours
*e*	3.916	0.316	4.257	0.671	1.209	4.431	2.856	1.459	0.928	2.365	8.068
$\bar{r}$	1.988	1.485	3.091	1.601	1.676	2.833	2.546	1.600	1.441	1.813	6.178
Blur	0.287	0.506	0.264	0.465	0.276	0.278	0.373	0.299	0.321	0.296	0.237
UIQM	1.478	1.038	1.540	1.094	1.425	1.563	1.385	1.293	1.207	1.375	1.594
DFAD	0.427	0.677	0.308	0.636	0.377	0.283	0.403	0.491	0.583	0.399	0.257

**Table 6:** Running time of the different methods (unit: s). ‘-’ indicates the result is not available. The best and the second-beat performers are shown in red and blue, respectively.

Method	HFM	DAUT	WWPF	UShape	OGO-ULAP	PCFB	SDGE	LANet	PUIENet	DeepWav	Ours
256×256	0.275	0.707	0.255	0.026	0.060	0.071	0.333	0.026	0.003	0.032	0.262
640×480	0.944	-	0.874	-	0.296	0.328	0.072	0.161	0.012	0.173	1.330
1800×1295	6.953	-	4.343	-	1.260	2.659	2.187	1.355	-	1.111	11.804

**Table 7:** Average quantitative results of different methods on the Figure 12. The best result is shown in red.

Method	GDCP	MR	CEEF	CAP	Ours
*e*	1.318	0.234	1.452	0.556	2.294
$\bar{r}$	1.752	0.850	2.286	0.947	6.668
DFAD	0.507	0.864	0.343	0.686	0.341

## Data Availability

The data used in this study are publicly available from the UCCS, UIQS, and UIEB datasets as cited in the paper.

## Abbreviations

The following abbreviations are used in this manuscript:

EFCR	Enhanced Layers Fusion and Color Restoration
PTC	Pixel-based Transmission Computation
MELF	Multiple-Enhanced-Layers Fusion
TCR	Transmission-driven Color Restoration
PTE	Pixel-based Transmission Estimation
DCP	Dark Channel Prior
AHE	Adaptive Histogram Equalization
WLS	Weighted Least Squares
UCCS	Underwater Color Correction Set
UIQS	Underwater Image Quality Set
UIEB	Underwater Image Enhancement Benchmark
CLAHE	Contrast-Limited Adaptive Histogram Equalization
UIQM	Underwater Image Quality Measure
DFAD	Dark Channel Prior-Free Assessment of Dehazing
